# Impact of HIV and chronic kidney disease comorbidities on hepatitis C treatment choices, drug–drug interactions and hepatitis C cure

**DOI:** 10.1007/s11096-020-00994-6

**Published:** 2020-02-25

**Authors:** Salamat Ali, Tofeeq Ur-Rehman, Eleri Lougher, David Mutimer, Mashhood Ali, Vibhu Paudyal

**Affiliations:** 1 Department of Pharmacy, Quaid-i-Azam University, Islamabad, Pakistan; 2 Abertawe Bro Morgannwg University Health Board, Princess of Wales Hospital, Swansea, UK; 3 Liver and Hepatobiliary Unit, University Hospitals Birmingham, Birmingham, UK; 4 Department of Gastroenterology, Pakistan Institute of Medical Sciences, Islamabad, Pakistan; 5 School of Pharmacy, University of Birmingham, Birmingham, B15 2TT UK

**Keywords:** Chronic kidney disease, Clinical outcomes, Drug–drug interactions, Hepatitis C, HIV, Human immuno-deficiency virus

## Abstract

*Background* Human immunodeficiency virus (HIV) co-infection and chronic kidney disease add challenges to hepatitis C virus treatment. *Objective* To conduct a comparative study of treatment choices, drug–drug interactions and clinical outcomes in hepatitis C mono-infected patients, or those with HIV or chronic kidney disease comorbidities. *Setting* Hepatitis C treatment centers of West Midlands England, United Kingdom. *Method* An observational study was conducted analyzing datasets of all hepatitis C patients that were referred to a large tertiary liver unit in the West Midlands, UK between July 2015 and January 2018. Patients aged ≥ 18 years with diagnosis of hepatitis C alone or co-infected with HIV or comorbid with chronic kidney disease were eligible. *Main outcome measures* The treatment choices, relevant potential drug–drug interactions and sustained virologic response 12 weeks post end of treatment were assessed. *Results* Out of 313 patients, 154 (49.2%) were hepatitis C mono-infected, 124 (39.6%) hepatitis C/HIV co-infected and 35 (11.2%) were hepatitis C/chronic kidney disease comorbid. There were 151 (98.1%) of hepatitis C mono-infected, 110 (88.7%) of hepatitis C/HIV and 20 (57.1%) of hepatitis C/chronic kidney disease patients treated with 1st line regimens. Significantly more patients who had co-morbidity with either HIV or chronic kidney disease were prescribed 2nd line regimens (8.1% and 37.1% respectively), compared to patients with hepatitis C mono-infection (1.9%) (*P* value < 0.05). Comorbid patients (12.1% of HIV and 25.8% of chronic kidney disease) were more likely to required drug–drug interactions advice (grade 5) than hepatitis C mono-infected (1.8%). Higher cure rates were observed in hepatitis C mono-infected (95.33%), hepatitis C/HIV (96.1%) compared to hepatitis C/chronic kidney disease patients (90.3%). *Conclusion* This study shows that treatment pathways permitting access to individual treatment adjustments in accordance with comorbidities and with consideration of drug–drug interaction in a multi-disciplinary team, provides successful outcomes in hepatitis C patients co-morbid with HIV or chronic kidney disease.

**Electronic supplementary material:**

The online version of this article (10.1007/s11096-020-00994-6) contains supplementary material, which is available to authorized users.

## Impacts on practice

- With treatment adjustments in accordance with comorbidities and with consideration of drug–drug interaction, it is possible to achieve high cure rates for hepatitis C patients who have HIV or chronic kidney disease.
- Input from multi-disciplinary team in decision making for co-morbid hepatitis C infection is imperative in achieving desirable treatment outcomes.

## Introduction

Comorbidities substantially impact on the progression and treatment of hepatitis C [1]. Globally, an estimated 71 million people have chronic hepatitis C (CHC) [2]. In the United Kingdom (UK), about 160,000 people have CHC with injecting drug use (IDU) as the primary (~ 90%) mode of transmission [3]. The UK’s Government hepatitis C action plan for England, identifies the need to scale-up treatment with new antiviral drugs if the burden of CHC is to be managed. Treating hepatitis C patients along with the comorbidities presents a considerable challenge for the health care providers [4, 5].

Certain comorbidities in CHC-infected patients may affect treatment outcomes, impair health related quality of life (HRQOL) [6, 7], and increase mortality associated with liver complications [8, 9]. There are two co-morbidities particularly important in clinical practice when treating hepatitis C, namely, chronic kidney disease (CKD) and human immunodeficiency virus (HIV) infection. CKD patients are vulnerable to contracting hepatitis C during dialysis [10], if the procedure is carried out using poor sterilization practices. In the context of the UK, the number of patients with co-morbidity of HCV and CKD is significant and includes mainly patients who undergo frequent travel to South Asia or Africa and who receive dialysis treatment whilst on holidays [11–14].

Likewise, the co-morbidity of HIV is prevalent, particularly in Injecting drug users (IDUs) [15]. Liver associated disease such as hepatitis C is a major reason of morbidity and mortality in HIV‐coinfection [16]. Also, HCV infection is being reported with increase in number among men who have sex with men (MSM) across Europe and the USA [17]. A Swiss cohort study has concluded that HCV infection accelerates the progression of HIV disease [18]. In HCV mono-infected patient, the progression of disease to cirrhosis takes almost 30 years in comparison to 15 years in co-infected patients [19].

The introduction of direct acting antivirals (DAAs) in 2011, and the further development of pan-genotypic DAAs, has provided highly effective and tolerable HCV drug regimens with cure rates greater than 95% [20]. However, in clinical practice, special considerations are required when prescribing DAAs for HCV/HIV co-infected patients due to concomitant medications and a potential increased risk of drug–drug interactions [21, 22]. Similarly, in HCV/CKD comorbidity, owing to accelerated development of hepatic complications and complex drug regimens, personalized patient care is critical [18, 23, 24]. It is thought that a multi-disciplinary team (MDT) approach inclusive of a prospective assessment of potential drug–drug interactions (DDIs) (prior to commencing HCV therapy), improves clinical outcomes and cure rates [25, 26].

In the UK, during 2016, twenty two Hepatitis C Operational Delivery Networks (ODNs) were launched to simplify patient access pathways to HCV testing and treatment services. The model encompasses a ‘hub and spoke’ approach with access to treatment being regulated by the hub that supports a specialised multi-disciplinary team to facilitate services at both the hub and at the spoke sites. This specialist team receives referrals of complicated, comorbid cases and ensures prospective evaluations and decision making for individualised patient care [27].

Alongside the implementation of the hepatitis C ODN within the UK, The National Health Service England (NHSE) introduced a hepatitis C treatment run rate card that sets clear prescribing guidelines for the hepatitis C regimens based on regimen prices. The guidelines stated that 90% of patients had to receive first line treatment, and that the use of second or third line treatment required review and approval from NHSE. As a result of the implementation of these guidelines, the MDT team were encouraged to make changes to the patients existing co-prescribed medication in order to avoid DDIs and comply with the run rate card, in preference to prescribing second or third line regimens.

### Aim of the study

This study aimed to explore the treatment choices; taking into account the prospective identification of DDIs by specialist pharmacist and to compare clinical outcomes, in HCV mono-infected patients, and HCV patients with HIV or CKD comorbidities.

### Ethics approval

The University Hospitals Birmingham (UHB) research and development committee reviewed this study and classified this as a service evaluation, hence not requiring full ethical submission. Necessary approval was then sought from the HR and the Clinical Audit Registration and Management System (CARMS-14077).

## Method

An observational study was undertaken. Datasets of all HCV monoinfected patients and with HIV/CKD comorbidities that were referred to a large tertiary Liver Unit in the West Midlands, UK, between July 2015 and January 2018 were analyzed.

Patients aged ≥ 18 years with diagnosis of hepatitis C alone or co-infected with HIV or comorbid with CKD were eligible for this study. Pregnant females, children and HCV patient having comorbidities other than HIV or CKD were excluded. The relevant patients were identified through the database of hospital portal.

### Data collection and analysis

A data collection tool was designed and moderated amongst the team of researchers including a senior medical consultant, a specialist pharmacist and a statistician. The data extraction was carried out by the study researcher and validated by the specialist pharmacist. Demography, ethnicity, mode of acquiring infection (sexual contact, IDU/people who inject drugs (PWID), perinatal exposure, iatrogenic exposure and other [tattoos, needle piercing, unknown]), genotype, baseline HCV viral load, HIV viral load, CD4 count, liver health status and data of monoinfected patients were collected from the tertiary liver unit databases and from the Clinical Portal (electronic interface containing patient records). Information detailing treatment history, medication prescribed was extracted from Clinical Portal/Prescribing, Information and Communication System (PICS) and referral forms (Appendix 1). The HCV regimen choice, along with any advice provided by the specialist pharmacist relating to the treatment choice, monitoring requirements and any requirements to change the patients existing medications were extracted from the correspondence found on clinical portal, minutes of the multi-disciplinary team meeting and PICS. Any missing information was pursued via request to the referring centre and follow up records were reviewed up to 12 weeks post end of treatment date.

Non-invasive transient elastography (using Echosens touch 502C scanner) was used to generate a fibroscan score which was used to determine the non-cirrhotic/cirrhotic status of each patient. A fibroscan score of 11.5 kPa or above was regarded as cirrhotic liver for the purpose of the study.

Follow up and treatment outcomes were assessed based on HCV viral load measured at beginning of treatment, at end of treatment (ETR) and 12 weeks post end of treatment [12 weeks Sustained Virological Response (SVR)]. A viral load of < 12 IU/ml 12 weeks post end of treatment was considered as undetectable level and was regarded as a cure. The final cure rate was reported in terms of modified intention to treat % (mITT %; considering the successfully treated patients and excluding patients who did not commence the treatment, stopped treatment early, those who were lost to follow up or were awaiting SVR12). The assessment of DDIs was carried out by the specialist pharmacist using the hepatitis C drug–drug interaction checker; Liverpool University, the electronic medicine compendium (eMC) for each drug and their clinical expertise. A coding tool was used to code DDIs. This tool was checked for face and content validity by the British Hepatitis Pharmacist Group (BHPG) which includes consultant pharmacist and specialist pharmacists working within the field of Hepatitis (Appendix 2). The codes explain risk rating and action required for potential drug–drug interactions.

All the data sets (anonymised) were sought from IT department at the tertiary centre and stored on password protected computers at all times. Statistical analyses were performed using SPSSV.24 (SAS Institute, Cary, NC). Frequency distribution and descriptive statistics were applied to demographic and baseline parameters. The Pearson Chi square test was applied to assess any difference of demographic, baseline and end point variables among groups and *P* value of 0.05 or less were taken as statistically significant.

## Results

A total of 313 patients met the inclusion criteria, of those 154 (49.2%) were HCV monoinfected, 124 (39.6%) HCV/HIV co-infected and 35 (11.2%) were HCV/CKD comorbid. The mean (SD) age was 51.9 (11.1) years. Two hundred and thirty four (74.8%) were male and 180 (57.5%) were white. Genotype 1a was the most prevalent 113 (36.1%) followed by genotype 3/3a—110 (35.1%). A total of 102 (32.6%) patients were PWIDs. Overall, one hundred and ninety two (61.3%) were non-cirrhotic while cirrhosis was diagnosed in 121 (38.7%) patients (Tables 1, 2).

**Table 1:** Demographic characteristics of patients; HCV, co-infected HCV/HIV and HCV/CKD (n = 313)

Demographic characteristics	Total (n = 313) n (%)	HCV (n = 154) n (%)	HCV/HIV (n = 124) n (%)	HCV/CKD (n = 35) n (%)
*Gender*				
Female	79 (25.2)	50 (32.5)	16 (12.9)	13 (37.1)
Male	234 (74.8)	104 (67.5)	108 (87.1)	22 (62.9)
*Age (years)*				
Mean (SD)	51.9 (11.1)	50.3 (12.4)	46.10 (10.4)	59.23 (10.6)
*Ethnicity/race*				
Asian or Asian British	47 (15.0)	32 (20.8)	3 (2.4)	12 (34.3)
White	180 (57.5)	56 (36.4)	104 (83.9)	20 (55.6)
Black, African, Caribbean or black British	21 (6.7)	7 (4.5)	11 (8.9)	3 (8.6)
Mixed or multiple ethnic group	2 (0.6)	1 (0.6)	1 (0.8)	–
Other ethnic group	5 (1.6)	5 (3.2)	–	
Prefer not to say	58 (18.5)	53 (34.4)	5 (4.0)	–
*Mode of infection*				
Heterosexual/MSM	10 (3.2)	–	8 (6.5)	2 (5.7)
IDU/PWID	102 (32.6)	13 (8.4)	80 (64.5)	9 (25.7)
Iatrogenic/non-occupational	31 (9.9)	8 (5.2)	4 (3.2)	19 (54.3)
Not known	170 (54.3)	133 (86.4)	32 (25.8)	5 (14.3)
*Genotype*				
1/1a	113 (36.1)	39 (25.3)	63 (50.8)	11 (31.4)
1b	50 (16.0)	29 (18.8)	13 (10.5)	8 (22.9)
2	5 (1.6)	3 (1.9)	2 (1.6)	–
3/3a	110 (35.1)	70 (45.5)	26 (21.0)	14 (40.0)
4/4d	33 (10.5)	12 (7.8)	19 (15.3)	2 (5.7)
5a	2 (0.6)	1 (0.6)	1 (0.8)	–
*Baseline viral load*^a^				
Very low viremic	14 (4.5)	6 (3.9)	8 (6.5)	–
Low viremic	37 (11.8)	11 (7.1)	22 (17.7)	4 (11.4)
Moderate viremic	140 (44.7)	83 (53.9)	42 (33.9)	15 (42.9)
High viremic	122 (39.0)	54 (35.1)	52 (41.9)	16 (45.7)
*Liver disease status*				
Non cirrhotic	192 (61.3)	68 (44.2)	102 (82.3)	22 (62.9)
Cirrhotic	121 (38.7)	86 (55.8)	22 (17.7)	13 (37.1)

*HIV* human immunodeficiency virus, *CKD* chronic kidney disease, *SD* standard deviation, *IDU* injecting drug user, *PWID* patient who inject drugs, *MSM* men who have sex with men

^a^Viral load is represented as; very low viremic = less than 8000 IU/ml, low viremic = 8001–20,000 IU/ml, moderate viremic = 20,001–800,000 IU/ml, and high viremic = greater than 800,000 IU/ml

**Table 2:** Patient’s characteristics, treatment choices and outcomes (n = 313)

Characteristics (N = 313)	HCV (n = 154) n (%)	HCV/HIV (n = 124) n (%)	HCV/CKD (n = 35) n (%)	*P* value
*Genotype*				
1a	39 (25.3)	63 (50.8)	11 (31.4)	< 0.001*
1b	29 (18.8)	13 (10.5)	08 (22.9)	
2	03 (1.9)	02 (1.6)	–	
3/3a	70 (45.5)	26 (21.0)	14 (40.0)	
4/4d	12 (7.8)	19 (15.3)	02 (5.7)	
5	01 (0.6)	01 (0.8)	–	
*Tx experience and liver status*				
Non-cirrhotic/naïve	45 (29.2)	73 (58.9)	17 (48.6)	< 0.001*
Non-cirrhotic/pre-treated	23 (14.9)	29 (23.4)	05 (14.3)	
Cirrhotic/naïve	39 (25.3)	14 (11.3)	10 (28.6)	
Cirrhotic/pre-treated	47 (30.5)	08 (6.5)	03 (8.6)	
*Tx choices*				
1st line	151 (98.1)	110 (88.7)	20 (57.1)	< 0.001*
2nd line	03 (1.9)	10 (8.1)	13 (37.1)	
3rd line	–	02 (1.6)	–	
Deferred/med change req.	–	02 (1.6)	02 (5.7)	
*Tx outcomes*				
Not commenced treatment	01 (0.6)	12 (9.7)	02 (5.7)	
Stopped early, lost to FU	02 (1.3), 01 (0.6)	02 (1.6), 07 (5.6)	02 (5.7), 00	
Completed treatment	150 (97.4)	103 (83.1)	31 (88.6)	
SVR 12 achieved	129 (83.8)	84 (67.7)	26 (74.3)	< 0.001*
ETR achieved/awaiting SVR12	14 (9.1)	15 (12.1)	02 (5.7)	
SVR 12 not achieved (failed/relapsed)	07 (4.5)	04 (3.2)	03 (8.6)	
mITT % cure rate	95.3%	96.1%	90.3%	

*HCV* hepatitis C virus, *HIV* human immunodeficiency virus, *CKD* chronic kidney disease, *Tx* treatment (1st line, 2nd line, 3rd line are in accordance with NHSE hepatitis C treatment run rate card at the time of treatment), *SVR* sustained virological response, *FU* follow up, *modified ITT* modified intention to treat analysis; representing; Tx of HCV patients was approved at MDT but not commenced and ongoing treatments awaiting 12 week SVR

*Pearson Chi square test shows a significant difference (*P* value 0.05 or less) of the variable among treated groups

**Fig. 1:**
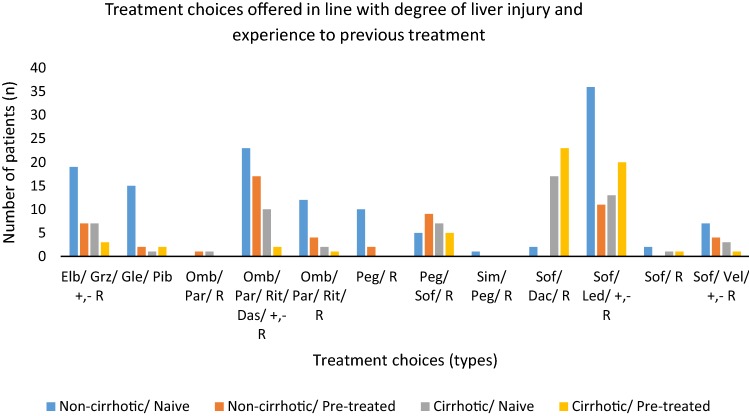
Frequency of treatment choices considering liver injury and treatment experiences. *Elb* elbasvir/grisepravir, *Sof/Led* sofosbuvir/ledipasvir, *Gle/Pib* glecaprevir/pibrentasvir, *Omb/Par/Rit/Das* ombitasvir/paritapravir/ritonavir/dasabuvir, *R* ribavirin, *Peg* pegylated interferon, *Sim* simprevir, *Led* ledipasvir, *Vel* velpatasvir

In treatment choices, sofosbuvir (Sof)/ledipasvir (Led) ± ribavirin (R) was the most (n = 37) prescribed combination of DAAs for non-cirrhotic/treatment naive patients ombitasvir (Omb)/paritapravir (Par)/ritonavir (Rit)/dasabuvir (Das) ± R was the second most prescribed combination (n = 23) and elbasvir (Elb)/grisepravir (Grz) ± R being the third most prescribed combination (n = 19) for this group of patients. Seventeen of the non-cirrhotic/treatment experienced patients received Omb/Par/Rit/Das ± R. There were 17 of cirrhotic/treatment naïve patients who were prescribed Sof/daclatasvir (Dac) ± R, while in cirrhotic/treatment experienced patients, Sof/Led ± R was prescribed to 20 patients. Figure 1 shows the range of treatment combinations prescribed.

The treatment choices were markedly influenced by genotype of HCV and appropriate selection of treatment regimen by MDT sustained individualized care. Resultantly, the patients with G1a were prescribed Sof/Led/+, − R (n = 55), Omb/Par/Rit/Das/+, − R (n = 30) and Elb/Grz/+, − R (n = 21). Similarly, patients having G3 infection were prescribed Sof/Dac/R (n = 42) Glecaprevir (Gle)/Pib (n = 18) and Sof/Velpatasvir (Vel)/+ , − R (n = 15).

### Clinical outcomes

A total 151 (98.1%) of HCV monoinfected, 110 (88.7%) of HCV/HIV and 20 (57.1%) of HCV/CKD patients were treated with 1st line HCV treatment in line with NHSE recommendations. Significantly more patients who had co-morbidity with either HIV or CKD were prescribed 2nd line regimens (n = 10, 8.1% and n = 13, 37.1% respectively), compared to patients with HCV monoinfection (n = 3, 1.9%) (*P* value < 0.05). Two (1.6%) of HCV/HIV patients were treated with 3rd line regimens. Two cases were deferred in each of HCV/HIV and HCV/CKD groups as change in concomitant medication was required (Table 2).

**Fig. 2:**
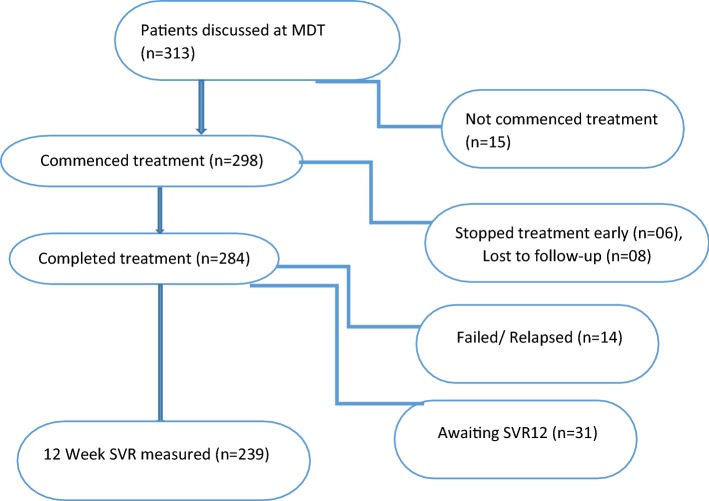
Flow diagram showing the treatment outcomes of the study cohort. *MDT* multidisciplinary team, *SVR* sustained virologic response

Of the total 313 patients, 239 (76.4%) achieved 12 week SVR and 31 (9.9%) achieved ETR and were awaiting 12 week post-treatment evaluation at the end of the study. Fourteen (4.5%) failed/relapsed, 6 (1.9%) discontinued treatment early, 8 (2.6%) were lost to follow-up and 15 (4.8%) did not progress to commence treatment beyond MDT decision. The overall mITT % cure rate was 95.1% (Fig. 2 and Table 2).

When compared HCV/CKD co-morbid (n = 28, 90.3%) patients, a greater percentage of patients with HCV who were monoinfected or co-infected with HIV, achieved mITT % (95.3% of HCV monoinfected and 96.1% of HCV/HIV respectively). The treatment choices, treatment outcomes and mITT % analyses are detailed in Table 2. There was no significant difference in treatment response in different ethnic groups; baseline viral load and SVR12 were similar in all ethnic groups (Table 2).

**Fig. 3:**
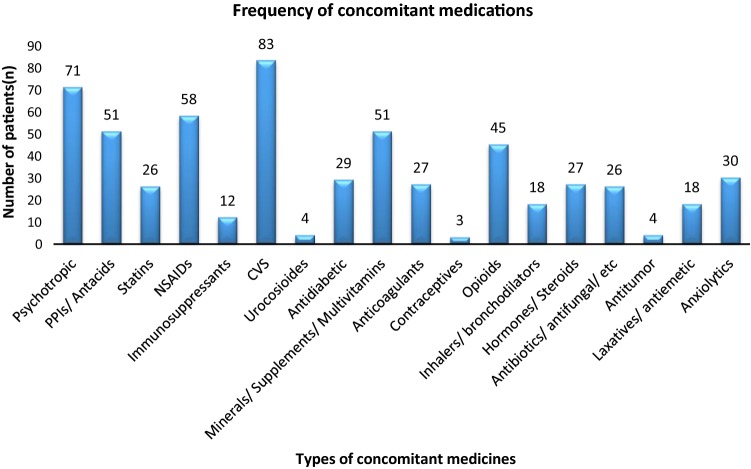
Frequency of concomitant medication prescribed along with hepatitis C medicines. *PPIs* proton pump inhibitors, *NSAIDs* non-steroidal anti-inflammatory drugs, *CVS* cardiovascular system drugs

Overall, the concomitant medications with potential DDIs were cardiovascular medicines 83 (26.4%), psychotropic medications 71 (22.7%), acid suppressants 51 (16.2%) [Including lansoprazole, omeprazole, ranitidine], statins 26 (8.3%) [Including atorvastatin, rosuvastatin, simvastatin, pravastatin, fluvastatin] and immunosuppressants (3.8%) (Fig. 3).

The median (IQR) number of concomitant medicines per patient were 2 (1–4) in HCV monoinfected, 3 (2–5) in HCV/HIV (excluding the HIV medications) and 8 (4–9) in HCV/CKD patients. The potential DDIs with concomitant medications were identified in a total 21 (13.6%) of HCV monoinfected patients, 56 (45.2%) of HCV/HIV medication and 19 (54.3%) of HCV/CKD medication (Table 3).

**Table 3:** Treatment choices, concomitant medication and prospective management of potential drug–drug interactions (DDIs)

Diagnosis (n = 313)	Treatment line/choices			Concomitant medication		Potential DDIs	Risk rating and interventions (%)		Outcomes measures n (%)				
	1st line n (%)	2nd line n (%)	3rd line n (%)	Median (IQR)	Min–max	Patients n (%)	Grade 1, 2	Grade 3, 5	Failed, relapsed	Tx stopped early, LOF	SVR12*	ETR achieved	mITT % cure rate
HCV (n = 154)	151 (98.1)^a^	3 (1.9)	–	2 (1–4)^b^	0–12	21 (13.6)^c^	76.2,10.4	11.6,1.8^d^	07 (4.5)	2 (1.3), 1 (0.6)	129 (83.8)	14 (9.1)	95.3
HCV/HIV (n = 124)^	110 (88.7)^a^	10 (8.1)	2 (1.6)	3 (2–5)^b^	0–16	56 (45.2)^c^	24.2,30.6	10.4, 12.1, 22.7 (gr. 4)^d^	04 (3.2)	2 (1.6), 7 (5.6)	84 (67.7)	15 (12.1)	96.1
HCV/CKD (n = 35)^	20 (57.1)^a^	13 (37.1)	–	8 (4–9)^b^	0–13	19 (54.3)^c^	17.1,28.6	28.5,25.8^d^	03 (8.6)	2 (5.7), 00	26 (74.3)	02 (5.7)	90.3

*DDIs* drug–drug interactions, *SD* standard deviation, *MDT* multidisciplinary team, *SVR 12* sustained virologic response at week 12 post treatment, *LOF* loss of follow up, *HCV* hepatitis C virus, *HIV* human immunodeficiency virus, *CKD* chronic kidney disease. *Gr* grade, rating of DDI interventions are in accordance to validation of British Hospital Pharmacist Group (BHPG), *mITT* modified intention to treat analysis [representing; Tx of HCV patients was approved at MDT but not commenced and ongoing treatments awaiting 12 week SVR (3 patients in HCV/HIV, 2 patients from HCV/CKD)]

A *P* value < 0.05 was taken significant. All parameters with superscript ^a, b, c, d and *^ are significantly different among groups. Note: Two cases were deferred in each of HCV/HIV and HCV/CKD group as change in concomitant medication was required

^ represents comorbidities

**Fig. 4:**
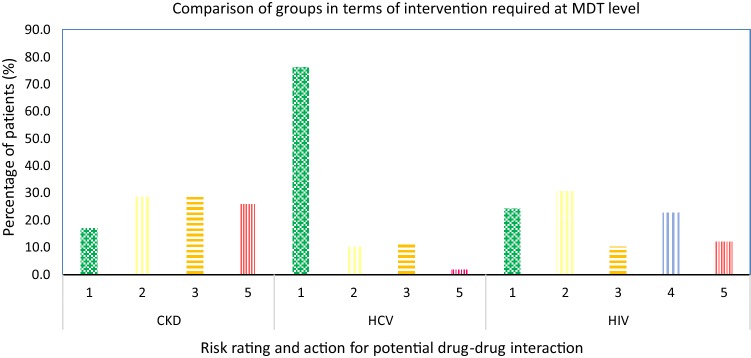
Prospective assessment of DDIs and interventions advised by multidisciplinary team (MDT). *HCV* hepatitis C virus, *CKD* chronic kidney disease, *HIV* human immunodeficiency virus. Risk rating and relevant interventions; 1 = no known drug–drug interaction/no action needed, 2 = advice on monitoring or counselling given by pharmacist, 3 = concomitant drug regimen changed, 4 = DDI with HIV/HCV regimen requiring a change in the HIV regimen or additional monitoring requirements and 5 = HCV drug regimen changed

The MDT prospectively assessed the risk of potential DDIs and advised interventions/action of grade 3 for 11.6%, 10.4% and 28.5% patients of HCV-monoinfected, HCV/HIV and HCV/CKD groups respectively. Similarly, interventions of grade 5 were advised for 1.8%, 12.1% and 25.8% patients of HCV mono-infected, HCV/HIV and HCV/CKD groups respectively. While, interventions of grade 4 were advised for 22.7% of HCV/HIV patients. There were more patients (76.2%) in HCV mono-infected group that did not require any modification in treatment choices compared to HCV/HIV (24.2%) and HCV/CKD patients (17.1%) (Fig. 4 and Appendix 2).

## Discussion

This study reports the selection and clinical effectiveness of the treatment choices in patients with HCV and concomitant comorbidities; specifically looking at the multi-disciplinary management of DDIs in hepatitis C treated patients in the UK. As per our results, treatment choices varied greatly due to the comorbidities. Treatment selection was made according to the individual patient’s circumstances as observed in the shifting of 1st line to 2nd in treatment choices. This was required more frequently in the HIV and CKD comorbid patients. In addition, the genotype, previous treatment and the presence of liver cirrhosis influenced the treatment choice significantly. The non-cirrhotic/treatment naive patients required fewer interventions and modifications when compared to cirrhotic/previously treated patients, who required greater interventions and treatment modifications.

This study confirms the importance of multi-disciplinary team (MDT) in decision making prospectively for HCV/HIV and HCV/CKD complicated cases [28]. This is demonstrated by results of mITT % cure rate 95.3%, 96.1% and 90.3% of HCV monoinfected, HCV/HIV and HCV/CKD patients respectively. A number of patients from both the HCV/HIV and the HCV/CKD groups however, either stopped the treatment early or were lost to follow-up. In HCV/HIV group, 2 (1.6%) patients stopped the treatment early and 7 (5.6%) were lost to follow-up, while in CKD, 2 (5.7%) patients stopped treatment early due to intolerable side effects. There were 4 (3.2%) treatment failures in HCV/HIV and 3 (8.6%) treatment failure in HCV/CKD comorbid patients. It’s worth noting that patients lost to follow up by the hepatology team in the HCV/HIV group, may have simply continued follow up by the HIV treating team.

At present, in the study setting, the access to DAA treatment for complicated cases such as those with HIV or CKD is dependent upon the referral to a specialist centre. The referral permits the specialist team to prospectively assess the patient’s clinical needs. This process includes an assessment of potential DDIs which contributes to the decisions surrounding the selection of the most appropriate HCV regimen. This model supports individualized patient’s care, the success of which is reflected in the high cure rates achieved. The findings of our study is provides addition in evidence to the currently available literature [29, 30] about the impact of pharmacist intervention and involvement as part of the MDT to address the DDIs associated with HCV management. While the burden of DDIs and co-morbidities in HCV have been previously illustrated [29], patient outcomes in relation to MDT interventions had not been explored before. Findings conforms with literature in other clinical areas which have demonstrated that inclusion of pharmacist in MDT with specific roles such as customization of treatment choices, dose modifications to improve adherence and prevention and management can help attain desirable treatment outcomes [31, 32].

DAA treatment choices conferred a success rate (mITT% cure) of 95.3% in HCV mono-infected and 96.1%, 90.3% for HIV and CKD comorbid patients respectively. Other comorbidities were common in the overall cohort and consequently the use of concomitant medication having potential DDIs was observed high with cardiovascular medicines 83 (26.4%) and psychotropic medications 71 (22.7%).

### Study strengths and limitations

This study has explored the role of multi-disciplinary team (MDT) in management of HCV and comorbid patients. Data was collected from a large tertiary hospital with adequate sample size for the planned statistical analyses of comparative outcome measurements. Validated and recognised data sources were used for all information on treatment choices and clinical outcomes.

This study has limitations. Firstly, the investigators were unable to access the data for mono-infected patients from the referring centres as only complicated cases are referred to the tertiary centre. Therefore, the PICS data (available at tertiary Liver unit) were used for the mono-infected patients. Secondly, the HIV or CKD and related treatment or management was ultimately under the control of respective departments, and thus the hepatitis C MDT relied on the respective departments to act on recommendations accordingly. Finally, this study was specific to the population served by the tertiary centre and may not reflect the whole population within the UK or further afield. In addition to the limitations listed, it’s worth noting at this stage that the study observation period was pre the introduction of Glecaprevir/pibrentasvir, thus patients with genotype 3 (G3) HCV and CKD were generally not referred to the MDT during the study period since the available treatment at that time contained a sofosbuvir back bone which is contraindicated in CKD. The population of CKD patient included in the study is small and predominantly included Genotype 1 (G1) and G3 HCV infections.

### Future research

This study highlights the role of a multi-disciplinary healthcare professionals’ input into the management of hepatitis C patients co-morbid with HIV or CKD. Based on our results, it is recommended that a future research work should include other co-morbid patients to explore the success of specialist hepatitis MDT regarding treatment outcomes in complex cases.

## Conclusion

This study shows that treatment pathways permitting access to specialised MDT care benefits HCV population who have HIV or CKD comorbidities. Individual treatment adjustments (in accordance with comorbidities, genotype and previous treatment experience) and consideration of drug–drug interaction by specialist pharmacist is likely to provide successful outcomes in HCV patients co-morbid with HIV or CKD.

Appropriate selection of DAAs to target HCV infection which presents with comorbidities can be enhanced through the inclusion and advice of a specialist pharmacist in a MDT setting.

### Electronic supplementary material

Below is the link to the electronic supplementary material.

Supplementary material 1 (PDF 329 kb)

## Data Availability

Technical appendix, statistical sheets, and dataset will be available from the Dryad repository and corresponding author at v.paudyal@bham.ac.uk.

## Appendix 2

See Table 4.

**Table 4:** Rating of risks, action and description for potential drug–drug interaction risk rating and action for potential drug–drug interaction

Classification (grade)	Action	Description
1	No known drug–drug interaction/no action needed	Discussed at MDT and no potential DDI identified
2	Advice on monitoring or counselling given by pharmacist	Discussed at MDT. Advice given by pharmacist regarding additional monitoring requirements/counselling points
3	Concomitant drug regimen changed	Discussed at MDT. Advice given regarding changing/omitting concomitant medications (excluding HIV drug regimens)
4	DDI with HIV/HCV regimen requiring a change in the HIV regimen or additional monitoring requirements	Discussed at MDT. Advice given regarding changing HIV regimen prior to commencing the hepatitis C treatment or additional monitoring requirements as a result of drug–drug interactions between the HIV/HCV regimens
5	HCV drug regimen changed	Discussed at MDT. Advice given regarding deviating from 1st line HCV regimen (in accordance with the current NHSE Run rate card at the time of treatment) due to drug–drug interactions

*MDT* multidisciplinary team, *DDI* drug–drug interactions, *HIV* human immunodeficiency virus, *HCV* hepatitis C virus, *NHSE* National Health Services England
