# Supplementary material for: Impact of HIV and chronic kidney disease comorbidities on hepatitis C treatment choices, drug–drug interactions and hepatitis C cure
Source: Int J Clin Pharm. 2020 Feb 25;42(2):515–26. doi: 10.1007/s11096-020-00994-6 (PMC7192872; doi:10.1007/s11096-020-00994-6)
Supplement: Supplementary file 1 — Supplementary material 1 (PDF 329 kb) [file 11096_2020_994_MOESM1_ESM.pdf]

Supplementary file: Genotypes and relevant frequency of treatment choices

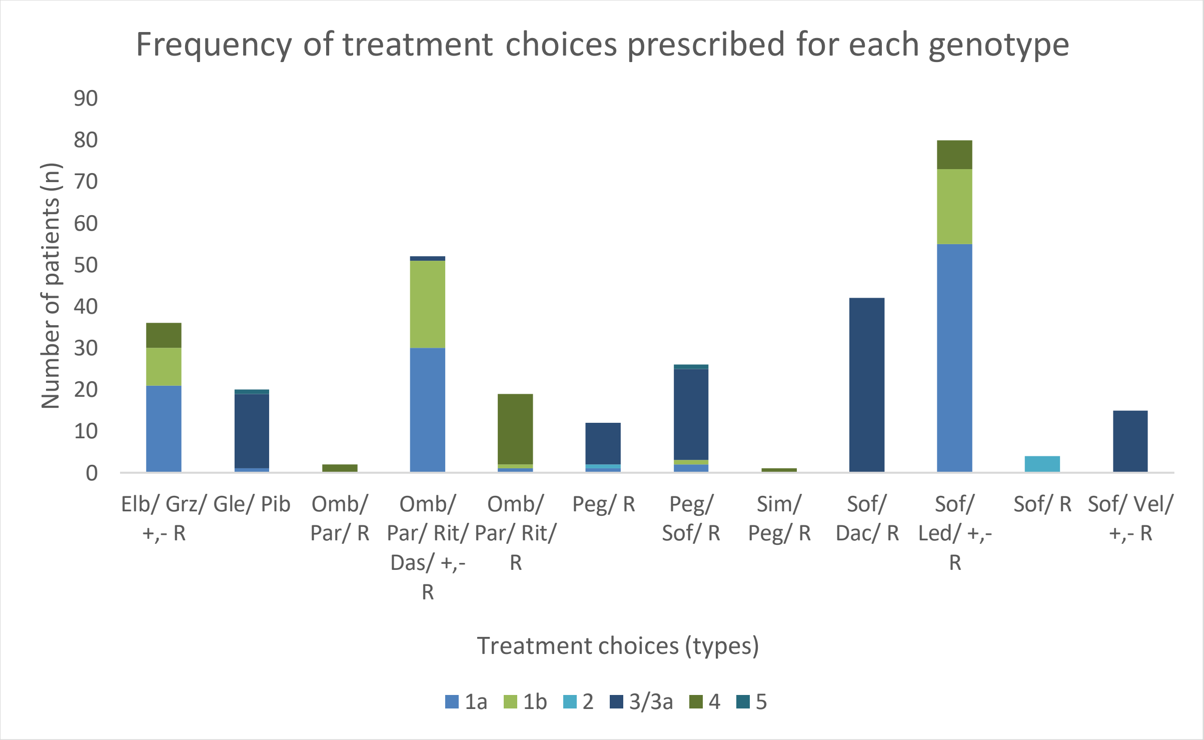

Abbreviations: Elb= Elbasvir/ Grisepravir, Sof/ Led= Sofosbuvir / Ledipasvir, Gle/ Pib= Glecaprevir/ pibrentasvir, Omb/ Par/ Rit/ Das= Ombitasvir/paritapravir/ritonavir/ dasabuvir, R= ribavirin, Peg= pegylated interferon, Sim= simprevir, Led= Ledipasvir, Vel= velpatasvir
